# Dentistry in France

**Published:** 1853-10

**Authors:** 


*Dentistry in France.*—We give the following as illustrative of Dental practice, and the mode of getting it in France. It appears to be a perfect contrast to the success of our American Dentists in that country.

With such men, however, as Drs. Brewster, Evans and others, who are prosecuting the profession so successfully in Europe, there is no danger but that the Profession itself will be sustained and advanced:

"Paris has long been the paradise of American dentists. Dr. Brewster, an intelligent Connecticut Yankee, made a splendid fortune here, which he is now worthily enjoying; and his successor, a young gentleman from Philadelphia, is amassing one still more rapidly. But let them beware of attempting to practice their art in any of the Provincial cities. The Provincials, cherishing their decaying stumps, hold dentistry and chloroform in equal horror, as the following narrative will prove. A well dressed stranger recently arrived in the ancient and famous city of Orleans. He went to the Frere-Directeur of the Ecoles Chretiennes, and representing himself as a dentist, stated that he intended to stop some time at Orleans to practice his profession, and, in order to make himself known was ready to examine the teeth of all children in his schools, and to do what was necessary gratuitously. As he produced documents amply proving his respectability and competency, and as he suggested that the parents of the children should be present at any operations he might perform, the Frere gave his consent. The next day he went to a school in the rue de l'Universite, and examined the mouths of the children. Having ascertained that several had teeth which required drawing, he asked the consent of such parents as were present to the operation, and on receiving a reply in the affirmative he extracted the teeth. Other children, influenced by the example of their companions, wished to have their teeth drawn also, affirming that their absent parents had consented to it, and the thing was done. The total number of children was about 400, and the dentist extracted from them 200 teeth. When the children went home with bloody mouths and faces bound up, the parents of many of them began to suspect that there had been foul play. In conversing with one another they became excited, and when it became known that nearly two hundred children had been operated on, the most extravagant rumors were spread.

The rumors gave rise to a panic after a while, and parents rushed in dismay to the different schools, and demanded that their children should be instantly given up to them. Some mothers seized large sticks, and threatened violence if their children were not immediately restored. A crowd of both sexes went to the Maison de la Visitation, and as some hesitation was shown in giving up the children, they broke open the doors and carried them off. At the school of Sainte-Croix the sisters of charity endeavored to convince them that their fears were unfounded; but they cried, 'The man is coming, give us up our children!' Similar scenes took place at the house of the sisters of St. Paul, at St. Marceau and St. Laurent. Even the boarding schools of M'lle. Manguin and M'lle. Grammont were evacuated, and as to the schools of the Freres of the Doctrine Chretienne not a single pupil was left in them. The mob next went to the Asylum, in the cloitre St. Pierrele-Puellier; and though a commissary of police employed all his efforts to convince them that there was no reason whatsoever for their terror, they carried off all the children.

They also attempted to cause the Ecole Normale to be evacuated, but M. Monvel, the director, firmly resisted them. The next day the agitation continued, and the most alarming reports spread. One was that a child who had eleven teeth extracted had died, but it is scarcely necessary to say that this was perfectly false. The departmental commissary and the commissaries of police employed all means in their power to dissipate the ridiculous fears of the parents, and it was hoped that they would eventually succeed. By their request the unfortunate dentist left the city. Had he fallen into the hands of the people at the moment the panic was at its height, he would no doubt have been torn in pieces.

Yours, JONATHAN

[*Correspondence of the Boston Post.*

